# Surgical Clipping of a Ruptured Distal Anterior Inferior Cerebellar Artery Aneurysm: A Technical Note

**DOI:** 10.7759/cureus.18688

**Published:** 2021-10-11

**Authors:** Zaid Aljuboori, Samer S Hoz, Zahraa F Al-Sharshahi, Dale Ding, Norberto Andaluz

**Affiliations:** 1 Neurological Surgery, University of Louisville School of Medicine, Louisville, USA; 2 Neurosurgery, Neurosurgery Teaching Hospital, Baghdad, IRQ; 3 Neurosurgery, University of Cincinnati Medical Center, Cincinnati, USA

**Keywords:** distal saccular aica aneurysm, distal artery aneurysms, microsurgical clipping, aneurysm, aica

## Abstract

Aneurysms of the distal anterior inferior cerebellar artery (AICA) are uncommon. They can present with subarachnoid hemorrhage (SAH), cerebellopontine angle syndrome, or a combination of the two. We describe the technique and nuances of microsurgical clipping of a ruptured distal AICA aneurysm using a retrosigmoid approach.

After performing the craniotomy, the AICA was exposed in a distal to proximal fashion and the aneurysm and the proximal parent vessel were identified. After establishing proximal control, a clip was placed across the neck of the aneurysm to obliterate it while maintaining flow within the parent vessel. Finally, the flow within the parent vessel was confirmed and the final clip position was checked to ensure that it was not compressing any of the cranial nerves in the vicinity. The aneurysm was completely obliterated, and the parent vessel remained patent.

Distal AICA aneurysms are rare and challenging to treat. The retrosigmoid approach is commonly used to treat these aneurysms. Careful planning, which includes studying the vascular anatomy and the aneurysm characteristics, and proficient execution of the procedure can increase the safety and improve outcomes of surgical clipping of these aneurysms.

## Introduction

The anterior inferior cerebellar artery (AICA) is a relatively uncommon location for intracranial aneurysms, with an incidence of 0.0003% to 0.5%, accounting for < 1-2% of all intracranial aneurysms [1]. In 1948, the first successful surgical treatment of an AICA aneurysm was performed by Schwartz et al. through a left retrosigmoid approach [2]. In 1966, the first Cooperative Study of Intracranial Aneurysms and Subarachnoid Hemorrhage was published, describing 6368 aneurysms, only two of which were located at the AICA [3].

The majority of AICA aneurysms occur at the meatal segment, with the dorsolateral branch of the artery being the most frequently involved [4]. Distal artery aneurysms are exceedingly rare. AICA aneurysms may present acutely with subarachnoid hemorrhage (SAH), or with recurrent cerebellopontine angle-related symptoms in larger aneurysms, such as unilateral sensorineural hearing loss, speech impairment, disequilibrium, tremor, or loss of motor control. This has been added to the introduction, or with a combination of the two. Open surgical techniques such as direct clipping, trapping, wrapping, ligation, en-bloc resection (associated with an AICA-fed arteriovenous malformation (AVM)), and endovascular therapy are amongst the available treatment options [4-9]

Due to the rarity of distal AICA aneurysms and their intricate microvascular architecture, their microsurgical clipping poses a challenge to the neurosurgeon and is case-specific. The purpose of this article is to discuss our experience using the retrosigmoid approach to clip a ruptured distal saccular AICA aneurysm in a 55-year-old patient with SAH.

## Technical report

**Figure 1:**
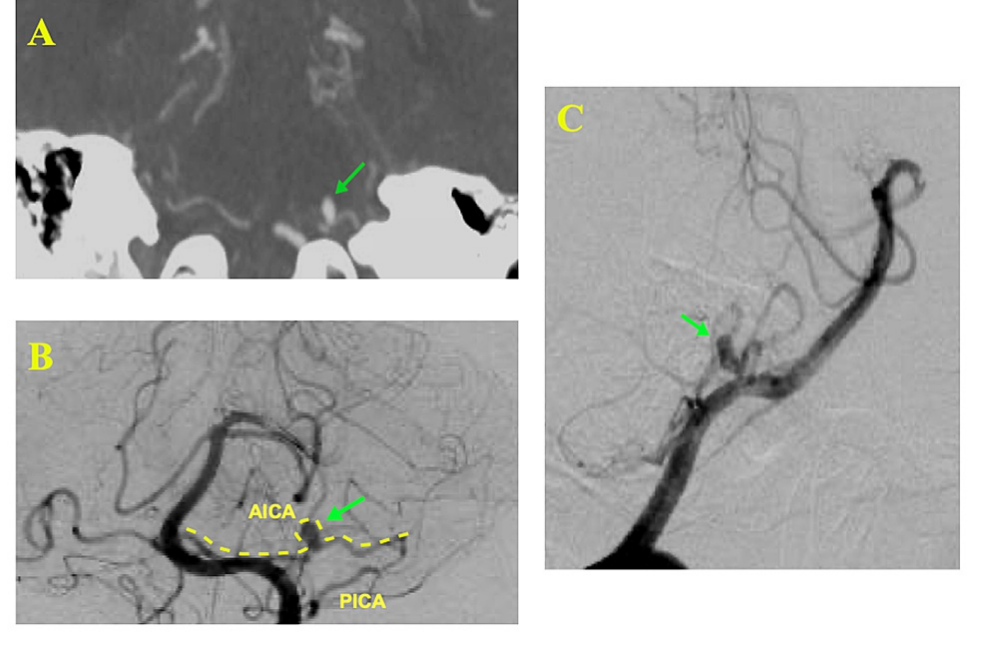
CT angiogram and diagnostic cerebral angiogram images show (A) AICA aneurysm in coronal view (arrow), (B) AICA aneurysm in AP view (arrow), (C) AICA aneurysm in lateral view (arrow). AICA: anterior inferior cerebellar artery; PICA: posterior inferior cerebellar artery; AP: anteroposterior

A 55-year-old female presented with a Hunt and Hess grade II, modified Fisher grade IV SAH. Cerebral angiography showed a left-sided distal saccular AICA aneurysm (Figure 1). The aneurysm was considered unfavorable for endovascular treatment by our team because it had a wide neck, which indicated the need for either stent or balloon-assisted coiling. Both options were not feasible because of the need for the use of dual antiplatelet, which can increase the risk for rebleeding. In addition, the AICA caliber was too small to accommodate a balloon and coiling microcatheter, and the distal location of the aneurysm and the acute angle of the AICA take-off indicated a potential difficulty in navigating a microcatheter with increased risk for catheter herniation. Therefore, the patient underwent a left-sided retrosigmoid craniotomy with clipping of the aneurysm. 

**Figure 2:**
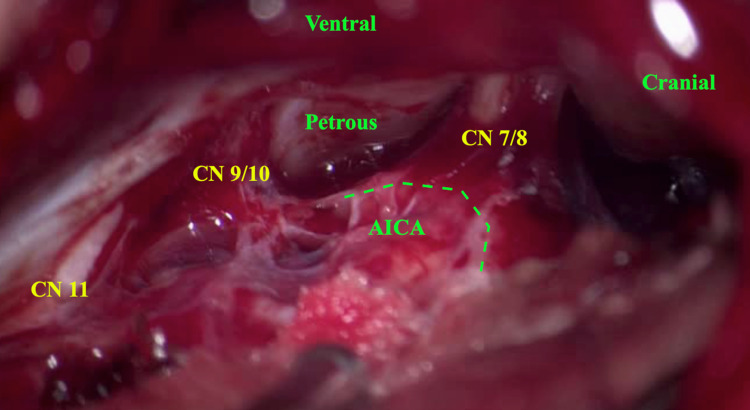
Microscopic picture [50x] shows the anatomical structures including the AICA (green dotted line) as seen through the retrosigmoid approach. AICA: anterior inferior cerebellar artery; CN: cranial nerve

After intubation, the patient is placed in a lateral position with the head placed in a Mayfield-Kees head holder parallel to the floor and in flexion. Twenty degrees of reverse Trendelenburg position is applied to promote venous outflow. Neuromonitoring (somatosensory evoked potentials test (SSEP), motor evoked potentials (MEP), and cranial nerves 5-12), and indocyanine green (ICG) dye are important adjuncts. An S-shaped incision is made approximately 3cm posterior to the mastoid process, extending 2-3cm above the superior nuchal line down to the level of the tip of the mastoid process; incision planning, in this case, was based on the surgeon’s preference. According to our experience, this type provides efficient exposure. Then, subperiosteal dissection is carried in both the anterior and posterior directions to expose the mastoid process along with 6cm of the occiput behind. Next, a retrosigmoid craniotomy is performed extending between the transverse sinus superiorly, the sigmoid sinus anteriorly, 5cm posterior to the sigmoid sinus, and approximately 3cm above the foramen magnum inferiorly. The dura is then opened and cerebrospinal fluid (CSF) is released to achieve cerebellar relaxation (Figure 2).

**Figure 3:**
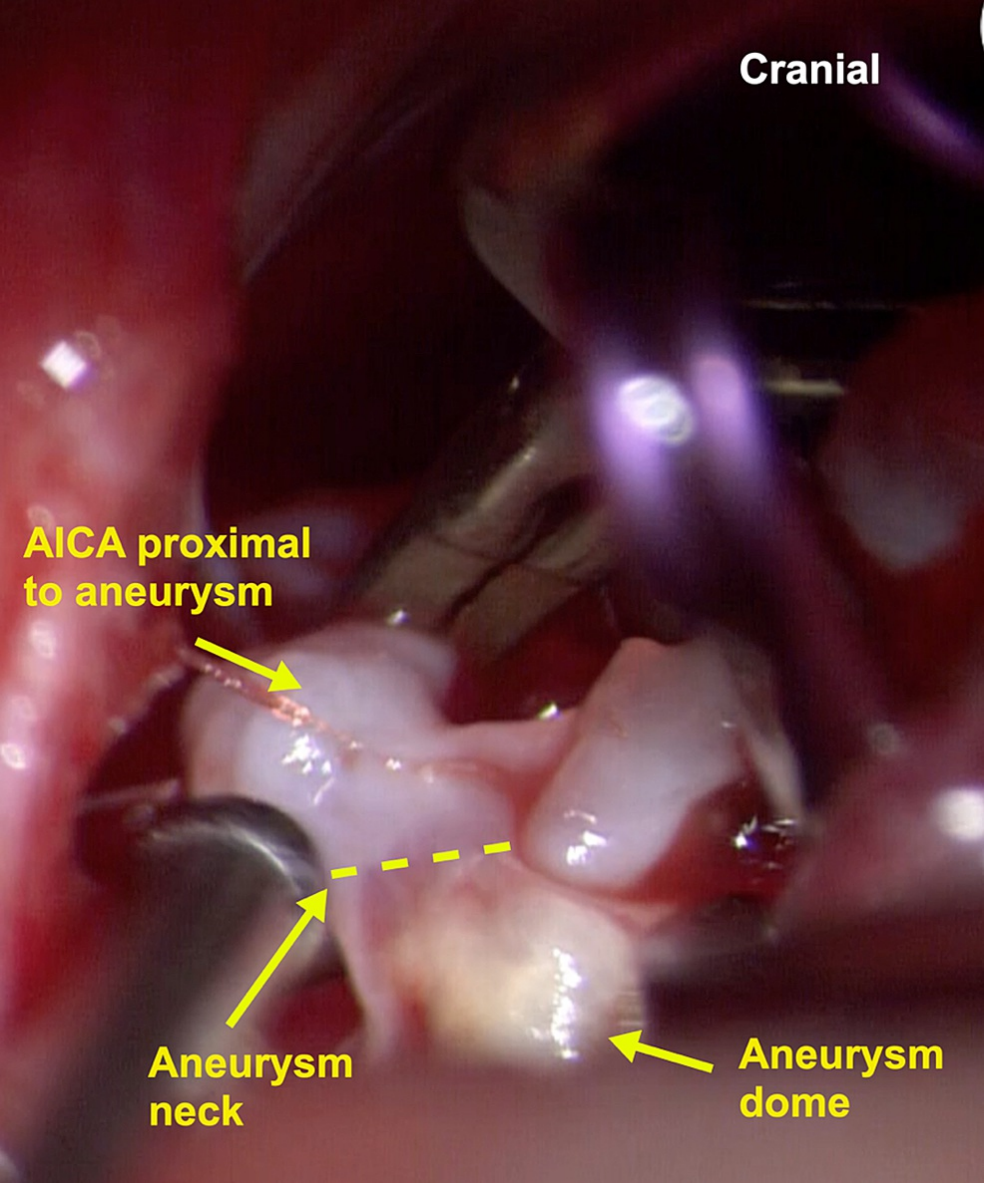
Microscopic picture [50x] shows the AICA, aneurysm neck (yellow dashed line), and the aneurysm dome. AICA: anterior inferior cerebellar artery

The AICA is then identified distally at the lateral edge of the petrosal surface of the cerebellum and followed proximally. We identified a part of the vessel that was suspicious for harboring the aneurysm due to the presence of a localized dark blood clot. Therefore, we avoided dissecting this area and identified a more proximal and healthier segment of the AICA at the level of the petroclival fissure, and applied a temporary clip (Figure 3).

**Figure 4:**
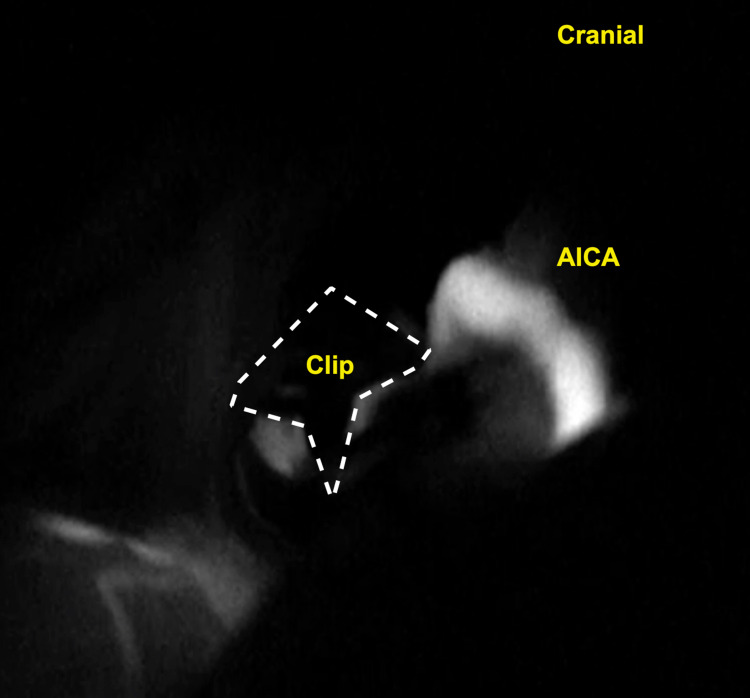
Microscopic picture of ICG shows patency of the AICA, and obliteration of the aneurysm. ICG: indocyanine green; AICA: anterior inferior cerebellar artery

The aneurysm is dissected, and it is determined whether or not the parent artery can be preserved. If the parent vessel can be preserved, a permanent clip is placed on the aneurysm neck, without kinking or constricting the parent artery. After removing the temporary clip, the blood flow is confirmed with a micro doppler and ICG (Figures 4). The dura and incision are closed in a multi-layered fashion, and the bone flap is replaced and secured in place with titanium screws and plates.

Postoperative course

**Figure 5:**
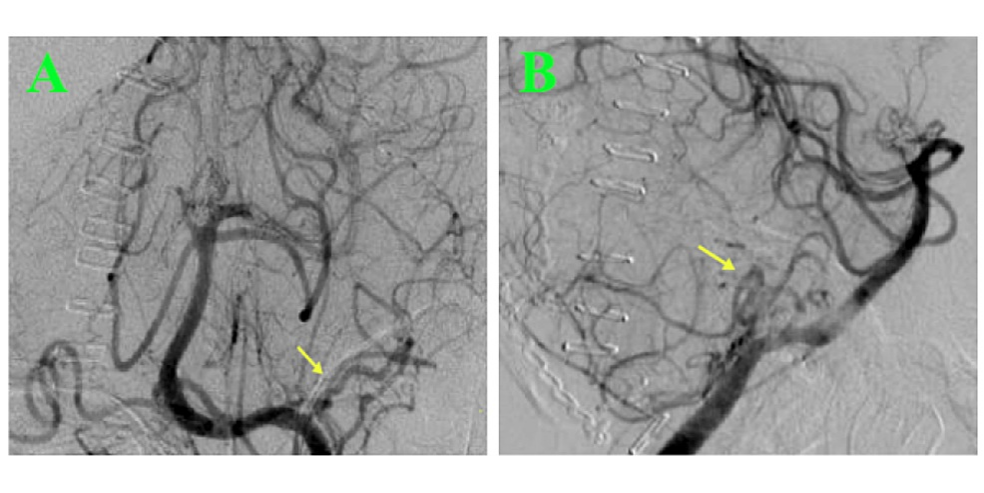
Post-clipping cerebral angiography images show obliteration of the AICA aneurysm (arrow) in the anteroposterior (A) and lateral (B) views. AICA: anterior inferior cerebellar artery

Postoperative angiography showed complete obliteration of the aneurysm with preservation of the parent vessel (Figure 5). The patient tolerated the procedure well and was extubated on postoperative day one. However, later during the hospital course, the patient developed pneumonia that was complicated by respiratory failure and sepsis with multiorgan failure resulting in the patient's demise. It is not known whether endovascular treatment would have altered the patient's course.

## Discussion

The AICA is a vessel of a variable origin, course, and supply. It is the most caudal of the three lateral branches of the basilar artery, typically originating from the middle or lower third of the vessel. It commonly originates as a single trunk, but a duplicated origin is a well-described variation. Leaving its origin, It then proceeds posterolaterally around the pons en route to the cerebellopontine angle. The main trunk divides into rostral and caudal trunks at the pontomedullary junction, more precisely at the exist of CN VII and VIII from the brainstem, supplying the upper and lower lips of the cerebellopontine angle and related structures. The vessel's path is intimately connected with the pons, lateral recess, foramen of Luschka, cerebellopontine fissure, middle cerebellar peduncle, and the petrosal surface of the cerebellum. 

**Figure 6:**
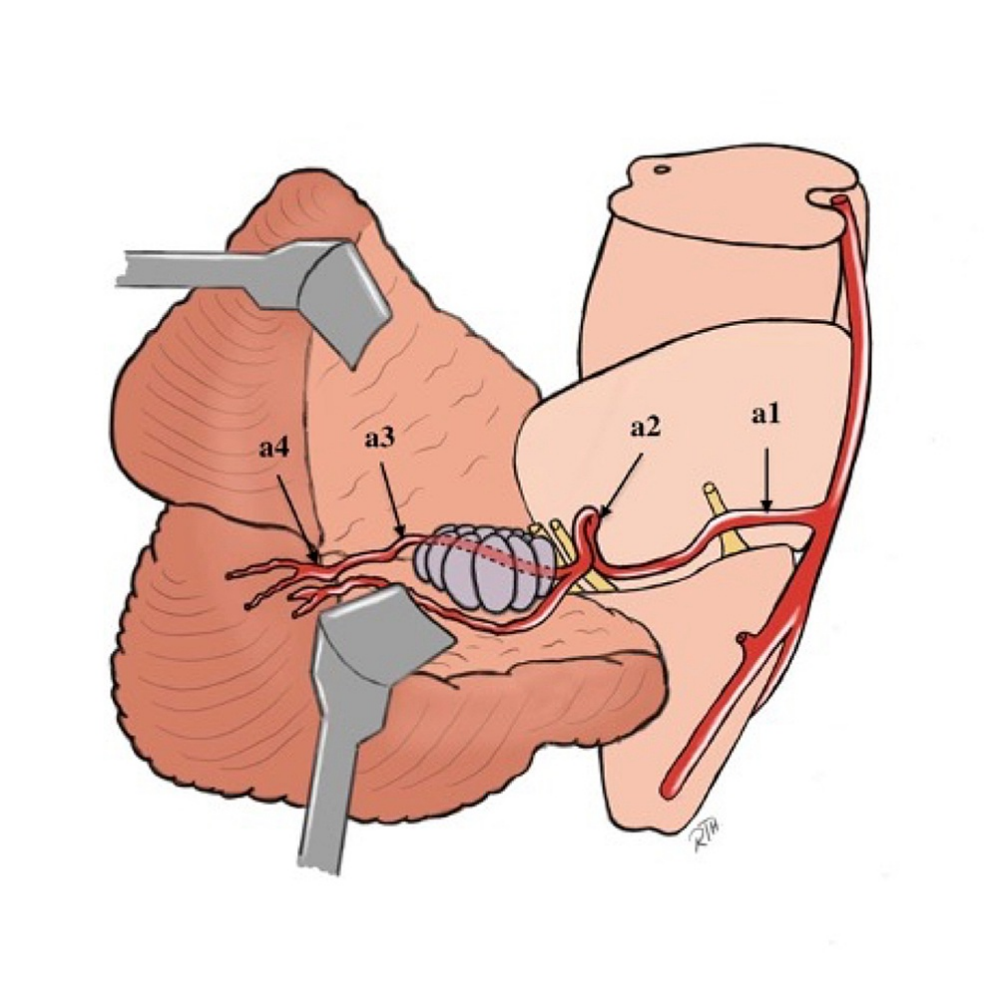
Artistic depiction of AICA course and segments. a1: anterior pontine segment; a2: lateral pontine segment; a3: flocculopeduncular segment; a4: cortical segment [Original illustration]

The AICA may be divided into four segments: the anterior pontine segment (Figure 6 a1) begins at the basilar artery and continues between the anterior pontine surface and the clivus, terminating at an upward-directed longitudinal axis drawn from the most prominent part of the medullary olive. This segment is intimately connected to the abducens nerve rootlets (CN VI). The lateral pontine segment (Figure 6 a2) begins proximal to the facial nerve and runs beneath, between, or above the facial (CN VII) and vestibulocochlear (CN VIII) nerves, terminating at the flocculus. Due to the segment's close relationship with the internal acoustic meatus, some authorities divide it into pre-meatal, meatal, and post-meatal subsegments. This segment gives rise to nerve-related branches, including the labyrinthine, which supplies CN VII and CN VIII as well as recurrent brainstem perforators and the subarcuate artery. As the name implies, the segment flocculopeduncular (Figure 6 a3) originates at the flocculus and continues superiorly or inferiorly to flocculus and the middle cerebellar peduncle en route to the cerebellopontine fissure. The cortical segment (Figure 6 a4) gives cortical branches to the petrosal cerebellar surface. During either endovascular or surgical treatment, the sacrifice of the AICA is only permitted after the segment distal to the origin of brainstem perforators [10,11]. Also, the AICA may be approached via the retrosigmoid, sub-temporal middle fossa, supra-tentorial, and infratentorial pre-sigmoid combined approaches, or translabrynthine approaches. The retrosigmoid approach is mainly used for AICA aneurysms. Occlusion of the proximal AICA may result in neurological deficits involving the cerebellum, the middle cerebellar peduncle, or the CNs V, VI, VII, and VIII. Ataxia of gait, dysmetria, nystagmus, motor weakness, trigeminal hypesthesia or dysesthesia, lateral gaze palsy, facial palsy, and hearing loss are all examples [4].

AICA aneurysms are extremely uncommon with fewer than 100 cases reported in the literature [12]. Typically, the term "distal" refers to AICA aneurysms arising “distal” to the origin of the AICA from the basilar artery; this is the definition used in this article. However, the literature is imprecise in this regard, describing a variety of nomenclatures and classification systems. Both endovascular and open microsurgical approaches have been identified as viable treatment options for these aneurysms. Numerous management options for distal AICA aneurysms have been described, and the procedure chosen is usually determined by aneurysm location, size, configuration, and patient condition [4,8]. Microsurgical intervention with direct clipping or trapping is commonly used as a first-line option, given the importance of permanently obliterating the aneurysm to eliminate the significant risks associated with re-bleeding [4-8]. One of the largest series reporting on the outcomes of AICA aneurysms was published by Gonzalez and colleagues who recommended the retrosigmoid approach for small and medium aneurysms involving the lower two-thirds of the clivus or the distal AICA [13].

Endovascular treatment remains an option, particularly for aneurysms with a favorable dome-to-neck ratio and those where microcatheter handling is feasible. Only ten cases of distal AICA aneurysms treated endovascularly have been published in the literature to date (14,15). In our case, we decided against endovascular therapy due to the configuration of the aneurysm and the surrounding vascular anatomy. The aneurysm was wide-necked (dome neck ratio 1.8), implying the need for balloon or stent-assisted coiling, neither of which was feasible considering the limited caliber of the vessel, which precluded microcatheter maneuvering or balloon insertion. Additionally, the vascular anatomy (parent vessel has a sharp angle proximal to the aneurysm) and the distal location of the aneurysm made maneuvering of the microcatheter more difficult without herniating the catheter's proximal portion into the basilar trunk. Also, the requirement for dual antiplatelet therapy, which carries a risk of hemorrhagic complications, made open microsurgical treatment the preferable option.

When accessing distal AICA aneurysms through the retrosigmoid approach, the following technical nuances can be advantageous: (1) It is necessary to identify the parent vessel distally and then proceed proximally, paying special attention to locating the aneurysm and then identifying the parent vessel proximal to the aneurysm to obtain proximal control. The distal-to-proximal dissection approach is ideal given the “distal” location of the aneurysm (parent vessel in the midline), and the small caliber of the AICA; (2) The surrounding neurovascular structures should be preserved, which may result in suboptimal exposure of the aneurysm neck; (3) Appropriate proximal control can be challenging for a variety of reasons, including the difficulty in dissecting proximal AICA segments, the tortuosity of its course, and the relatively frequent occurrence of anatomical variations at the AICA origin, such as bifid AICA and a common AICA-PICA origin trunk; (4) There is no universal agreement on the maximum duration of temporary clipping of vessels such as the AICA. As a result, it is critical to keep temporary clipping to a minimum. One possibility is to use a sequential temporary clipping technique, with the clip advancing more proximally towards the aneurysm. The latter technique may help to minimize the risk of brainstem ischemia from occluding the AICA perforators that supply the brain stem and cerebellum. (5) Temporary aneurysm trapping is typically indicated in cases of giant, difficult-to-dissect, or partially thrombosed aneurysms to facilitate designed clipping; a maneuver that is frequently difficult in cases of AICA aneurysms due to the narrow surgical corridor and the requirement for backflow to maintain perfusion of perforating branches. Additionally, in the event of an intraoperative rupture, continuous suctioning may be sufficient to allow visualization and control of the rupture point, depending on the amount of backflow; (6) Finally, special care should be taken to ensure that the final clip position does not compress nearby cranial nerves.

In light of the limited experience with distal AICA aneurysms, there is no consensus regarding the optimal treatment strategy. Therefore, an individualized approach should be pursued and the choice between the open microsurgical and endovascular approaches should take into account both the aneurysm and patient characteristics. 

## Conclusions

There is limited experience of distal AICA aneurysms due to their rarity. While the retrosigmoid approach is the most straightforward for these aneurysms, the deep surgical corridor with its narrow angle of view and the presence of critical neurovascular structures such as the brain stem and cerebellar perforators, make these aneurysms challenging for the neurosurgeon. However, a meticulous technique that takes the anatomical configuration of these aneurysms and the geometry of the surrounding neurovascular structures into account may result in an uneventful clipping with favorable surgical outcomes.
